# Functional Characterization of Spinocerebellar Ataxia Associated Dynorphin A Mutant Peptides

**DOI:** 10.3390/biomedicines9121882

**Published:** 2021-12-11

**Authors:** Andreas Lieb, Germana Thaler, Barbara Fogli, Olga Trovato, Mitja Amon Posch, Teresa Kaserer, Luca Zangrandi

**Affiliations:** 1 Institute of Pharmacology, Medical University of Innsbruck, 6020 Innsbruck, Austria; andreas.lieb@i-med.ac.at (A.L.); germana.thaler@i-med.ac.at (G.T.); barbara.fogli@i-med.ac.at (B.F.); olga.trovato@i-med.ac.at (O.T.); mitja.posch@student.i-med.ac.at (M.A.P.); 2 Center for Molecular Biosciences Innsbruck, Department of Pharmaceutical Chemistry, Institute of Pharmacy, University of Innsbruck, 6020 Innsbruck, Austria

**Keywords:** dynorphin, kappa opioid receptor, spinocerebellar ataxia, β-arrestin, G-protein, functional selectivity, biased agonism, ligand-directed signaling, TRUPATH

## Abstract

Mutations in the prodynorphin gene (*PDYN*) are associated with the development of spinocerebellar ataxia type 23 (SCA23). Pathogenic missense mutations are localized predominantly in the *PDYN* region coding for the dynorphin A (DynA) neuropeptide and lead to persistently elevated mutant peptide levels with neurotoxic properties. The main DynA target in the central nervous system is the kappa opioid receptor (KOR), a member of the G-protein coupled receptor family, which can elicit signaling cascades mediated by G-protein dissociation as well as β-arrestin recruitment. To date, a thorough analysis of the functional profile for the pathogenic SCA23 DynA mutants at KOR is still missing. To elucidate the role of DynA mutants, we used a combination of assays to investigate the differential activation of G-protein subunits and β-arrestin. In addition, we applied molecular modelling techniques to provide a rationale for the underlying mechanism. Our results demonstrate that DynA mutations, associated with a severe ataxic phenotype, decrease potency of KOR activation, both for G-protein dissociation as well as β-arrestin recruitment. Molecular modelling suggests that this loss of function is due to disruption of critical interactions between DynA and the receptor. In conclusion, this study advances our understanding of KOR signal transduction upon DynA wild type or mutant peptide binding.

## 1. Introduction

Spinocerebellar ataxia 23 (SCA23) is an autosomal dominant neurodegenerative disorder mainly characterized by progressive impairment of motor coordination and cerebellar ataxia associated with Purkinje cell death in the cerebellum [1]. SCA23-causing mutations have been reported in the prodynorphin gene (*PDYN*), coding for the precursor protein for the opioid neuropeptides α-neoendorphin, dynorphin (Dyn) A and B. Mutations are located predominantly in the *PDYN* region coding for DynA, which is highly conserved throughout species [2,3]. DynA mutations relevant for SCA23 pathophysiology, which result in a change of the coding sequence, include leucine at position 5 to a serine (L5S), arginine at position 6 to a tryptophane (R6W), and arginine at position 9 to a cysteine (R9C). These three DynA mutants display neurotoxic properties in cultured striatal neurons [2] and induce pathological pain when injected intrathecally into mice at femtomolar doses [4]. In addition, transgenic mice ubiquitously expressing DynA_R6W (the most toxic SCA23-associated DynA mutant) showed altered glutamatergic signaling, neuronal excitability, and motor performance [5], thus demonstrating the importance of this mutant for the initiation and progression of SCA23. To date, the cellular mechanisms underlying these neurotoxic effects have been attributed in part to a loss of affinity to kappa opioid receptors (KOR), increased peptide degradation resistance, and a switch from opioid to NMDA-receptor signaling [6]. Furthermore, increased peptide stability may result in protein aggregation and penetration of bilayer membrane leading to leakage and cellular dysfunctions [7,8,9].

The KOR is the main target of DynA in the central nervous system [10]. It is well established that DynA-mediated KOR activation plays an essential role in pain regulation, substance abuse disorders, and stress and anxiety [11,12,13]. Initially, it was hypothesized that all KOR-dependent effects resulted from G-protein-mediated signaling exclusively. However, the discovery that G-protein coupled receptors can generate G-protein independent signaling through β-arrestin recruitment (for review see [14]), where it functions as a scaffold for different signaling molecules [15,16], led to the conceptualization of the “ligand-directed signaling” theory [17]. The “ligand-directed signaling” theory, also known as “functional selectivity” or “biased agonism”, states that different ligands can bind and stabilize the receptor in alternative active conformations resulting in secondary messenger biased intracellular signaling profiles. β-arrestin recruitment has already been reported for KOR [18,19] with both small molecules [20] and endogenous agonists [21] displaying clear biased agonism.

At the cellular level, it has been shown that β-arrestin recruitment by KOR is a necessary event leading to desensitization [18], as well as KOR internalization [19]. In addition, it has been demonstrated that β-arrestin-dependent signal transduction is crucial for the induction of KOR-mediated side effects. Indeed, p38 MAPK phosphorylation, which is directly linked to β-arrestin recruitment in KOR signaling cascade [22], is responsible for the induction of a hyposerotoninergic state associated with depression-like and drug-seeking behaviour in rodents [23]. Furthermore, dysphoric-like behavior [24,25] can be observed as a typical drawback of KOR activation by small molecules.

The possibility of a biased signal may also arise from the G-protein signaling cascade itself. To date, more than 15 Gα subunits have been described and divided into four major families based upon structural and functional homology: G α_s_, G α_i/o_, G α_q/11_, and Gα_12/13_. Specifically, KOR couples to G proteins of the G α_i/o_ family, which consist of two Go members, three Gi, Gz, two transducins (rod and cone), and gustducin [26]. KOR exhibits promiscuity for the members of G α_i/o_ family [27], raising the question as to whether DynA SCA23 mutants preferentially activate one G-protein subunit over another.

To our knowledge, there are no studies investigating the ability of DynA SCA23 mutants to recruit diverse G α subunits or β-arrestin. Therefore, a comprehensive analysis on the role of the SCA23 mutations on KOR receptor functionality is still missing. In this study, we took advantage of state of the art in vitro techniques to explore the signaling pathways activated by the most relevant DynA mutants involved in SCA23 aetiology.

## 2. Materials and Methods

### 2.1. Peptides

Wild type and mutated Dynorphin A peptides were synthetized and purchased from GenScript Biotech Corporation (#SC1208; Leiden, The Netherlands).

### 2.2. Molecular Biology

For [^35^S] Guanosine 5′-O-[γ-thio]triphosphate (GTP-γ-S) and G protein TRansdUcer PATHways (TRUPATH) experiments, the human OPRK cDNA (DNASU Plasmid repository HsCD00515607; Seiler et al., 2014) was cloned into pcDNA3.1 in fusion with the alpha7 nicotinic signal peptide and eGFP (N terminal on backbone) [28]. For parallel receptorome expression and screening via transcriptional output, with transcriptional activation following arrestin translocation (PRESTO-Tango) experiments, OPRK cDNA (DNASU Plasmid repository HsCD00515607; [29]) was cloned into OPRK1-Tango which was a gift from Bryan Roth (Addgene plasmid # 66462; http://n2t.net/addgene:66462; 10 December 2021; RRID:Addgene_66462; [30]). TRUPATH was a gift from Bryan Roth (Addgene kit #1000000163; [31]), pTetON-EGFP was a gift from Brad Zuchero (Addgene plasmid # 89453; http://n2t.net/addgene:89453; 10 December 2021; RRID:Addgene_89453; [32]), and pCDNA3.1(+)-CMV-bArrestin2-TEV was a gift from Bryan Roth (Addgene plasmid # 107245; http://n2t.net/addgene:107245; 10 December 2021; RRID:Addgene_107245).

### 2.3. Cell Culture

CHO cells stably transfected with human KOR receptors (hKOR-CHO) were courtesy of Prof. Liu-Chen (Temple University, Philadelphia, PA, USA). Cells were grown in Gibco™ Dulbecco’s Minimal Essential Medium (DMEM) supplemented with 10% FBS, Glutamax (100 μg/mL), penicillin/ streptomycin (100 μg/mL) and geneticin (G418, 400 μg/mL). HEK 293 cells (gift from Prof. Joerg Striessnig, University of Innsbruck, Innsbruck, Austria) were grown in DMEM supplemented with 10% FBS, Glutamax (100 μg/mL) and penicillin/streptomycin (100 μg/mL). Cell cultures were maintained in a Heracell™ 150i CO_2_ incubator at 37 °C and 5.0% CO_2_.

### 2.4. [^35^S] Guanosine 5′-O-[γ-thio]triphosphate Functional Assays for κ Opioid Receptors

Functional assays were conducted on hKOR-CHO cell membranes. Membranes were prepared in 50 mM Tris-HCl buffer pH 7.7. Cells were harvested by scraping the plates with a rubber policeman and were then centrifuged at 500× *g* for 10 min. The cell pellet was resuspended in Tris-HCl, homogenized with a Dounce homogenizer and centrifuged at 27,000× *g* for 15 min. The pellets were resuspended in Tris-HCl and homogenized through a 27G needle. The protein concentration of the homogenate was determined by Biuret reaction (RotiQuant Universal, Carl Roth) and stored at −70 °C until use.

To perform the GTPγS assay, membrane homogenates (10 μg/reaction tube) were diluted in HEPES buffer (20 mM) together with 10 μM GDP and 0.05 nM [^35^S]GTPγS (Perkin Elmer, MA, USA). The buffer containing the cell membranes was then incubated with increasing concentration of test peptide (total volume of 1 mL) for 60 min at 25 °C. Non-specific binding was determined using 10 μM unlabeled GTPγS (Sigma-Aldrich, Vienna, Austria). Samples were filtered over glass fiber filters (GF/B Whatman) and the bound radioactivity was measured by liquid scintillation counting as counts per minute (CPM).

### 2.5. β-Arrestin Recruitment Assay

For β-arrestin recruitment, the PRESTO-Tango assays were conducted in HEK293. On day 1, 20000 cells per well were seeded in a black 96-well plate with clear bottom. The following day (day 2), cells were transfected using 1:1:1:1 DNA ratio of tTA-hKOR (receptor):TEV-*β-arrestin:Tet-GFP:mCherry* (100 ng). PEI MAX (Polysciences, Inc., Warrington, PA, USA) diluted in OptiMEM (Gibco-ThermoFisher) was used at 2:1 PEI to DNA ratio. On day 3, peptides were diluted in DMEM and applied on transfected cells for overnight incubation. On the fourth day, GFP fluorescence intensity was measured using a Spark Tecan plate reader.

Results in the form of relative fluorescence units (RFU) were exported into Excel spreadsheets, and GraphPad Prism was used for analysis of data.

### 2.6. G-Protein Transducerome Assay

For investigation of different G-protein subunit activation, the TRUPATH assays were conducted in HEK293. On day 1, 25,000 cells/well were seeded in a white 96-well plate. The following day (day 2), cells were transfected using a 1:1:1:1 DNA ratio of receptor:*Gα-RLuc8:Gβ:Gγ-GFP2* (100 ng). PEI MAX (Polysciences, Inc., Warrington, PA, USA) diluted in OptiMEM (Gibco-ThermoFisher) was used at 2:1 PEI to DNA. Then, 48 h later (day 4) growth medium was carefully aspirated and replaced immediately with 60 µL of assay buffer (1× Hank’s balanced salt solution (HBSS) + 20 mM HEPES, pH 7.4), followed by a 10 µL addition of freshly prepared 50 µM coelenterazine 400a (Nanolight Technologies). After a 5 min equilibration period, cells were treated with 30 µL of drug for 30 min. Plates were then read in a Spark Tecan plate reader with 395 nm (RLuc8-coelenterazine 400a) and 510 nm (GFP2) emission filters, at integration times of 1 s per well. BRET ratios were computed as the ratio of the GFP2 emission to RLuc8 emission. GraphPad Prism was used for analysis of data.

### 2.7. Modelling

The NMR structure of hKOR-bound Dyn (PDB entry 2N2F; [33]) was combined with the cryo-EM structure of the mu opioid receptor (MOR) interacting with DAMGO (PDB entry 6DDE [34]), a synthetic peptidomimetic analogue of enkephalin, to model the active conformation of hKOR in complex with dynorphin residues 1-7. Please note that all residues after R7 in the Dyn NMR structure clashed with the MOR template, suggesting that the Dyn structure might not be a reliable template for these residues.

The first four amino acids (i.e., 1-YGGF-4) are shared between enkephalin and Dyn and backbone conformations were similar in both PDB entries. The static DAMGO model was therefore considered a better template for these residues than the NMR structure, which captures much more side chain flexibility and the binding conformation can thus not be unequivocally assigned.

Therefore, the Dyn NMR structure (PBD entry 2N2F, state 8; [33]) was aligned to DAMGO in the MOR-DAMGO complex (PDB entry 6DDE) in Molecular Operating Environment (MOE) version 2019.01.02 [35]. Residues 1 to 4 in DAMGO were merged with residues 5-7 in Dyn to create a new structural template for modelling of Dyn 1-7. This was used in addition to the MOR (PDB entry 6DDE) as a template to generate a homology model of the Dyn 1-7 peptide in complex with hKOR using the default settings in MOE. The final model was further refined by removing the negative charge at the carboxyterminus of R7 and the complex was prepared again with the Protein Preparation Wizard [36,37] in Maestro [38] Schrödinger version 2019.4 using the default settings, except that a restrained minimization was included.

Mutations were introduced with the Protein Builder tool in MOE, side chain rotamers were sampled and the rotamer with the lowest energy was kept. Figures were created using PyMOL version 1.8.0.0 [39].

## 3. Results

### 3.1. SCA23 DynA Mutant Exhibit Reduced hKOR Activation

Following receptor binding by agonists (e.g., DynA or DynB), hKOR initiates the dissociation of Gα subunit and G*β*γ subunits which then proceed to interact with various intracellular effectors [40]. To shed light on the ability of DynA_R6W to activate hKOR, we first examined the impact of this mutation on G-protein activation. To evaluate its agonist activity, we performed GTPγS experiments on hKOR cell membranes in comparison with the wildtype peptide (DynA_WT). 

**Figure 1:**
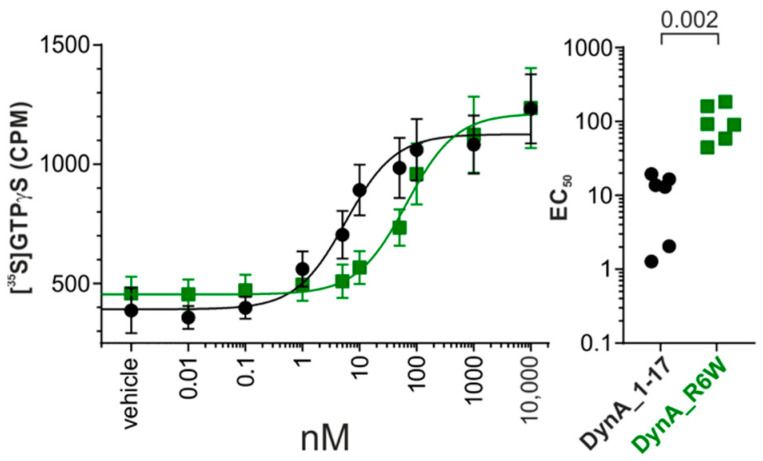
Dynorphin A (DynA)_1-17 and DynA_R6W peptides signal at human kappa opioid receptor (hKOR). Left: Dose response curve of [^35^S]GTPγS binding to membranes (10 μg) from cells expressing hKOR. Right: Summary showing the magnitude of the change in EC_50_ induced by DynA_1-17 and DynA_R6W peptides in each experiment. Data are shown as mean ± SEM from six independent experiments. Mann–Whitney test, *p*-values are indicated in the figure. CPM: counts per minute.

The dose response curve we obtained displayed significantly lower potency for DynA_R6W with an EC_50_ of 105 ± 23 nM compared to DynA_WT EC_50_ of 11 ± 3 nM (*p* = 0.002, Mann-Whitney) (Figure 1, Supplementary Table S1). Nevertheless, this mutation had no effect on the efficacy with both peptides capable of fully activating hKOR (DynA_WT:1179 ± 128 CPM Vs DynA_R6W: 1245 ± 164 CPM, *p* = 0.589, Mann–Whitney). These data indicate a partial loss of function in hKOR signaling that might be relevant to the SCA23 pathology.

### 3.2. G-Protein Transducerome Activation by DynA_R6W

Given the promiscuity of KOR for the members of G α_i/o_ family [27], we hypothesized that the SCA23 mutant DynA may couple different G-protein subunits compared to the wild type isoform. To test our hypothesis we took advantage of the TRUPATH biosensors, a suite developed to comprehensively profile GPCR coupling preferences [31]. In this experiment, only six members of the G α_i/o_ family were tested (α1, α2, α3, αoA, αoB, αZ), since the two transducins and gustducin are not expressed in the central nervous system.

**Figure 2:**
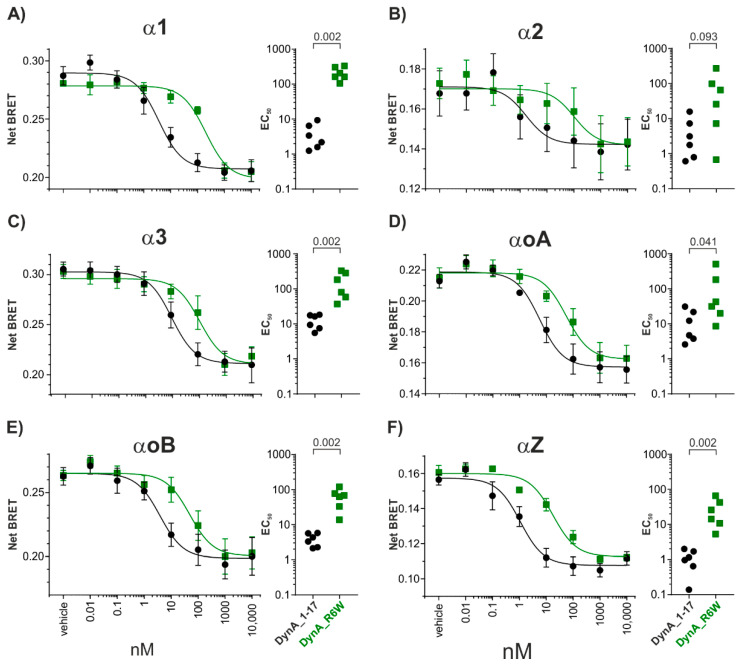
DynA_1-17 and DynA_R6W peptides transducerome at hKOR. (**A**–**F**) Left: Dose response curves of TRUPATH (BRET assay) in HEK293 cells transiently transfected with hKOR and the different Gα subunits. (**A**–**F**) Right: Summary showing the magnitude of the change in EC_50_ induced by DynA_1-17 and DynA_R6W peptides for each Gα subunit. Data are mean ± SEM. Mann–Whitney test, *p*-values are indicated in the figure.

The profile of hKOR and its endogenous agonist DynA shows similar G-protein activation between all the G α_i/o_ subunits tested. The EC_50_s were comparable to that of the GTPγS experiment (Figure 2A–F). Likewise, the SCA23 mutant DynA_R6W activates all G α_i/o_ subunits investigated to a similar extent as DynA_WT. However, as observed for the GTPγS assay, the potency is significantly reduced for α1, α3 αoB and αZ subunits (EC_50_ values are summarized in Supplementary Table S1, respectively; Figure 2A,C,E,F). These results are in line with those from the GTPγS experiment and indicate that the loss of potency is not specific for any of the G α_i/o_ subunits.

### 3.3. Investigating Signal Bias at hKOR

In the last decade, small molecule drug discovery of KOR activators as analgesics focused mainly on the development of biased agonists. The rationale for this is based on the fact that aversion, one of the main KOR-dependent side effects, is mediated by p38 MAPK phosphorylation [24,25,41], which in turn depends on the recruitment of β-arrestin [22]. Therefore, a number of compounds with these characteristics have been recently developed [20]. Moreover, it has been shown that endogenous opioid ligands too may retain biased agonist features [21]. For this reason, we investigated the G-protein and β-arrestin activation pattern of the DynA mutants (DynA_R6W, DynA_L5S and DynA_R9C) associated with SCA23 [2].

Since the previous experiment using the TRUPATH suite did not reveal differences in G α_i/o_ subunits by neither DynA_WT nor DynA_R6W application, we decided to assess G-protein activation only on the α3 subunit. 

**Figure 3:**
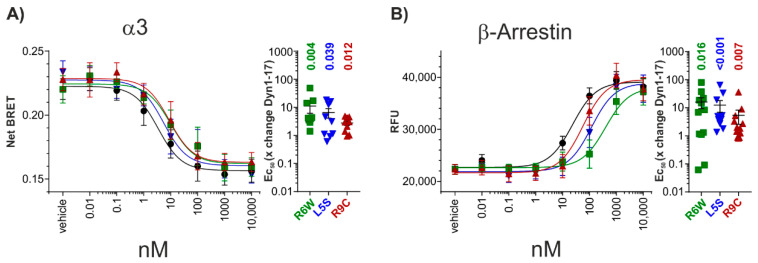
Signal bias investigation for DynA_1-17 (Black), DynA_R6W (green), DynA_L5S (blue), and DynA_R9C (red) peptides at hKOR. (**A**) Left: Dose response curve obtained from TRUPATH (BRET assay) measuring G-protein dissociation in HEK293 cells transiently transfected with hKOR and the Gα3 subunit. Right: Magnitude change in EC_50_ induced by DynA_1-17 SCA23 mutant peptides for Gα3 subunit in comparison to mean same day DynA_WT control. (**B**) Left: Dose response curve obtain from PRESTO-Tango assay measuring β-arrestin recruitment in HEK293 cells transiently transfected with hKOR. Right: Magnitude change in EC_50_ induced by DynA_1-17 and the SCA23 mutant peptides in comparison to mean same day control. Data are shown as mean ± SEM. Wilcoxon Signed Rank Test; *p*-values are indicated in the figure.

Investigation of G-protein signalling revealed that all the peptides induced a dose-dependent increase in G protein activity with nanomolar potency and comparable efficacy (Figure 3A). The SCA23 mutants display significantly lower potency, specifically, DynA_R6W 11 ± 5, DynA_L5S 7 ± 2, and DynA_R9C 3 ± 1 fold shift in comparison to the mean of same day DynA_WT control experiments (DynA_R6W: *p* = 0.004, DynA_L5S: *p* = 0.04, DynA_R9C: *p* = 0.012, Wilcoxon Signed Rank Test) (Supplementary Table S1). Next, we examined the signalling profile of these peptides using the β-arrestin recruitment assay PRESTO-Tango. In this assay, DynA_WT and the three mutant peptides also dose-dependently recruited β-arrestin with comparable efficacies and all three mutations also showed a decreased potency in comparison to DynA_WT (DynA_R6W 16 ± 7, DynA_L5S 13 ± 6, and DynA_R9C 5 ± 3, fold shift in comparison to the mean of same day DynA_WT control experiments (DynA_R6W: *p* = 0.016, DynA_L5S: *p* < 0.001, DynA_R9C: *p* = 0.007, Wilcoxon Signed Rank Test)) (Figure 3B, Supplementary Table S1). Together, these results indicate that all SCA23 mutants lose potency for G-protein and β-arrestin activation to a similar extent.

### 3.4. Molecular Modelling

**Figure 4:**
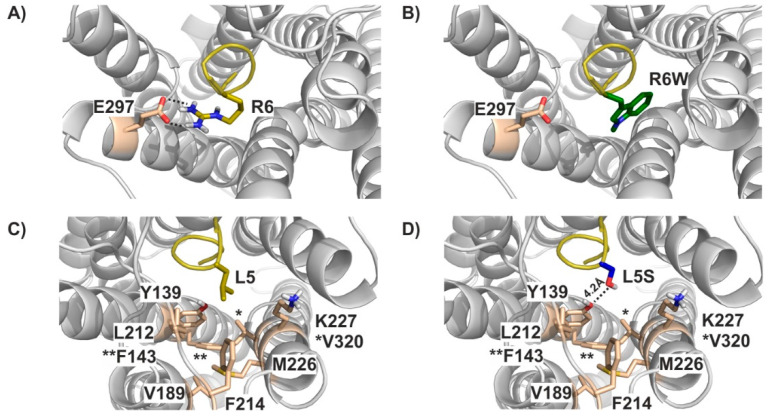
Molecular modelling of DynA_WT, DynA_R6W and DynA_L5S. DynA R6 forms an ionic interaction with E297 (**A**), which is disrupted in the R6W mutant (**B**). (**C**) Dyn L5 is involved in hydrophobic contacts with a range of non-polar KOR residues lining a lipophilic side pocket. Similarly, these interactions cannot be formed with the L5S mutant (**D**). * and ** indicate the position of V320 and F143, respectively.

In the next step, we aimed to elucidate the underlying mechanism on a molecular level. In the absence of an experimental structure of the hKOR-Dyn complex, we generated a model of hKOR bound to residues 1-7 of DynA. Please note that available templates only allowed us to model the first seven residues of DynA with sufficient confidence (please see Methods section for details). In this model, tyrosine Y1 and phenylalanine F4 in the 1-YGGF-4 motif, which are strictly conserved among endogenous ligands of the KOR, MOR, and delta opioid receptor (DOR) [42], superpose well with the corresponding substructures in other peptidomimetic and small molecule opioid ligands in experimental structures (Supplementary Figure S1) [34,43,44]. Intriguingly, arginine R6 forms an ionic interaction with glutamate E297 (Figure 4A) in our model. This residue position displays a high variability in the otherwise well conserved opioid receptor orthosteric binding pocket and has been implicated in mediating receptor selectivity [44]. Mutation of R6 to W (i.e., the R6W mutation) results in the loss of the charged interaction with this critical residue (Figure 4B), suggesting a considerable detrimental impact on DynA binding.

Similarly, the apolar side chain of leucine L5 extends into a hydrophobic cavity formed by residues Y139, F143, V189, L212, F214, M226, V230, and K227 (Figure 4C). Introduction of the serine hydroxy-group (Figure 4D) would not only disrupt hydrophobic contacts within that pocket, but also be energetically unfavorable due to placement of a polar moiety into a lipophilic environment. The latter could, at least partly, be compensated by interactions with the phenolic OH-group of Y139 or the side chains of K227 or E297. These groups are too far away from the L5S in our current static model (e.g., 4.2 Å between OH-group in S5 and Y139), but we would expect that only minor conformational re-arrangements would be required to form these hydrogen bonds, thus potentially explaining the less severe impact of L5S on DynA binding—and ultimately function—compared to the R6W mutation.

## 4. Discussion

In the present study, we examined the pharmacological characteristics of DynA mutants directly associated with SCA23 pathogenesis. 

The analyzed missense mutations were identified in four Dutch families displaying progressive gait and limb ataxia and they were all located in the *PDYN* part coding for the neuropeptide DynA. The three DynA mutants (R6W, L5S and R9C) display neurotoxic properties in cultured striatal neurons [2], and induce pathological pain when injected into mice. Among these, DynA_R6W showed the highest level of toxicity and non-opioid nociceptive activity [4]. In addition, the causality between the *PDYN* mutations and SCA23 was demonstrated in transgenic mice ubiquitously expressing DynA_R6W, which displayed impairment in their motor performance and neuropathological changes similar to those observed in SCA23 patients [5].

To date, the mechanisms underlying these effects remain unclear. An attempt to investigate how SCA23 mutants promote neurotoxicity was recently made by Smeets and colleagues [6]. Their study revealed an altered secondary structure of the peptides resulting in increased peptide stability, reduced KOR receptor affinity and a partial switch from opioid to NMDA-receptor signaling. Furthermore, the reduced solubility of DynA mutants and their capacity to penetrate bilayer membranes can induce pore formation and subsequent ion leakage through the membrane, a non-receptor mediated mechanism that may contribute to the neurotoxic effects [7,8,9,45]. However, a comprehensive investigation on the role of the SCA23 mutations on KOR receptor functionality was still missing.

With the intention of elucidating the effects of SCA23 mutants on hKOR, we performed GTPγS experiments using the mutant peptide most relevant for SCA23 etiology (DynA_R6W) and observed a partial loss of function in hKOR signaling. The decreased affinity of DynA_R6W for hKOR has been previously observed [6] and our findings demonstrate that this is reflected in receptor activation. To further elucidate the functional profile of DynA_R6W, we took advantage of a newly established BRET suite, which allows us to interrogate the hKOR transducerome (Gα subunits resolution) [31]. Similar to what we observed in the GTPγS experiments, DynA_R6W displayed a significantly reduced potency for the investigated Gα1, α3, αoB and αZ compared to wild-type DynA and a clear tendency for Gα2 and GαoA, suggesting that a differential G-protein subunit engagement is likely not playing a role in DynA_R6W neurotoxicity. Interestingly, endogenous DynA (DynA_WT) does not discriminate between different G α_i/o_ subunits in the central nervous system.

Growing evidence shows that different orthosteric agonists can stabilize distinct receptor conformations and activate distinct downstream signaling pathways [46], a phenomenon called functional selectivity or biased agonism [47]. It has been shown that endogenous opioid peptides can exhibit functional selectivity [21], thus we decided to explore the ability of SCA23 mutant peptides to activate G-protein and β-arrestin. In both assays, all SCA23 mutant peptides displayed a significant reduction in potency. 

To further understand how DynA_R6W is transducing signals through hKOR, we generated a molecular model of hKOR bound to the first seven DynA residues. This analysis revealed that the arginine in position 6 forms an ionic interaction with glutamate E297 on the transmembrane helix 6 (TM6). The R6W mutation disrupts the interaction with this residue, thus providing a good rational for the reduced DynA_R6W binding and functionality. The E297 residue is located on TM6, whose large conformational change is required for receptor activation [43]. Therefore, our data support the hypothesis that the E297 residue is critical for DynA binding to hKOR. The interaction with this specific residue may shape signal transduction by conferring distinct intracellular receptor conformations.

Many SCA subtypes share a common genetic cause, namely the expansion of CAG-repeat sequences in specific genes, and as a consequence abnormally long polyQ tracts in the proteins are encoded [48]. This results in a common toxic gain-of-function mechanism for the polyglutamine-containing protein, which leads to abnormal activation patterns, altered membrane targeting, enhanced activity and protein aggregation with nuclear and cytosolic inclusions [49], typical neuropathological hallmarks for SCA [50]. The genetic mutations analyzed in this study are of a different class. The investigated missense mutations involved in the pathogenesis of SCA23, on the contrary, lead to a loss-of-function for the main target of DynA, the hKOR, as shown by our results. However, such mutations are also responsible for increased DynA levels, protein aggregation and enhanced NMDAR signalling [6,7,8,9], causing a neurodegenerative phenotype frequently observed in SCA.

In conclusion, we were able to demonstrate that all DynA mutants significantly decrease their potency at hKOR for both G-protein and β-arrestin activation, and we speculate that such effect in DynA_R6W might involve an altered interaction with the E297 residue.

This study improves our understanding of how SCA23-associated DynA mutants transduce signal through hKOR. In addition, it provides structural insight on key protein residues involved in ligand affinity, potency and perhaps functional selectivity. However, further experiments investigating signalling networks in more detail, such as phosphoproteomic appraches, will be necessary in order to demonstrate the causal relationship between neurotoxic effects of DynA and specific molecular pathways.

## Supplementary Materials

The following are available online at https://www.mdpi.com/article/10.3390/biomedicines9121882/s1, Figure S1: Alignment of Dyn to other opioid receptor agonists, Table S1: Summary of GTPγS, TRUPATH and PRESTO-Tango experiments.
