# Supplementary material for: Functional Characterization of Spinocerebellar Ataxia Associated Dynorphin A Mutant Peptides
Source: Biomedicines. 2021 Dec 11;9(12):1882. doi: 10.3390/biomedicines9121882 (PMC8698333; doi:10.3390/biomedicines9121882)
Supplement: Supplementary file 1 [file biomedicines-09-01882-s001.zip › biomedicines-1485896-supplementary.pdf]

Supplementary Material

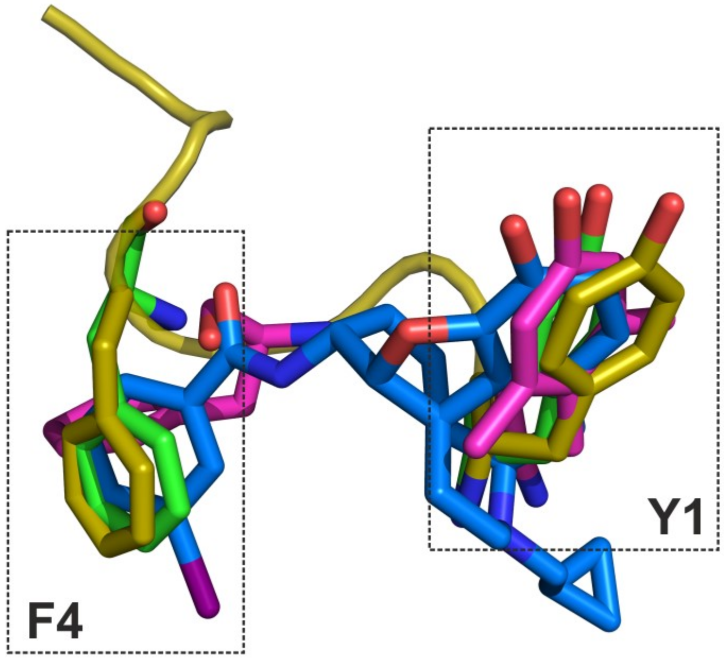

Figure S1: Alignment of Dyn to other opioid receptor agonists. The residues Y1 and F4 in the 1-YGGF-4 motif are strictly conserved among endogenous ligands of the KOR, MOR, and DOR. Alignment of Dyn (gold) to other opioid receptor agonists highlights superposition of these two residues with corresponding substructures in experimental receptor-ligand complexes. This suggests that our DynA-KOR model represents a biologically relevant binding mode. Green: DAMGO (synthetic enkephalin peptidomimetic, MOR, PDB entry 6DDE [34], blue: MP1104 (opioid agonist, KOR, PDB entry 6B73[43]), magenta: KGCHM07 (synthetic peptidomimetic agonist, DOR, PDB entry 6PT2 [44]. Please note that for clarity, only the relevant substructures of the peptidomimetic agonists are depicted.

Table S1: Summary of GTPγS, TRUPATH and PRESTO-Tango experiments. EC_{50} values for each peptide are shown as mean ± SEM (the number of experiments, the fold change (x-change) in comparison to DynA-WT (if applicable), and the p-value are in indicated). Experiments marked with # were performed on the same cell batch for comparison. * Mann-Whitney test vs. DynA-WT, § Wilcoxon Signed Rank Test

		DynA-WT (nM)	R6W (nM)	L5S (nM)	R9C (nM)
**GTPγS**		11±3 (6)	105±23 (6, 0.002*)	n.d	n.d
**TRUPATH**	α1	4±1 (6)	211±36(6, 0.002*)	n.d	n.d
	α2	5±2 (6)	78±42 (6, 0.093*)	n.d	n.d
	α3	12±2 (6)<br>7±2 # (9)	161±51 (6, 0.002*)<br>133±75 # (9, 11±5 x-change, 0.004§)	78±37# (9, 7±2 x-change, 0.039)	24±9# (9, 3±1, 0.012§)
	αoA	13 (6)	133±80 (6, 0.042*)	n.d	n.d
	αoB	4±1 (6)	62±15 (6, 0.002*)	n.d	n.d
	αZ	1±1 (6)	27±9 (6, 0.002*)	n.d	n.d
**β-Arrestin**		37±11# (12)	670±215# (12, 16±7 x-change, 0.016§)	236±65# (12, 13±6 x-change, <0.001§)	88±32# (12, 5±3 x-change, 0.007§)
